# MCID normalization: A methodological framework for harmonizing heterogeneous PROMs in hip arthroscopy research

**DOI:** 10.1002/jeo2.70568

**Published:** 2025-11-15

**Authors:** Nikolai Ramadanov

**Affiliations:** ^1^ Center of Orthopaedics and Traumatology, Brandenburg Medical School University Hospital Brandenburg an der Havel Brandenburg an der Havel Germany; ^2^ Faculty of Health Science Brandenburg Brandenburg Medical School Theodor Fontane Brandenburg an der Havel Germany

**Keywords:** hip arthroscopy, MCID, meta‐analysis, methodology, outcome harmonization, PROM

## Abstract

Hip arthroscopy (HAS) research employs a wide range of patient‐reported outcome measures (PROMs). This heterogeneity complicates cross‐study comparisons and meta‐analyses, as no gold‐standard PROM exists. The minimal clinically important difference (MCID) offers a clinically meaningful threshold of change, but methodological guidance on integrating heterogeneous PROMs through MCID has been lacking. Can heterogeneous PROMs in HAS be harmonized through MCID normalization? PROMs reported in 100 HAS studies were identified and ranked according to frequency of use. The most frequently reported MCID values from published validation studies were applied. Each PROM was then evaluated and normalized by dividing observed mean values and standard deviations by the respective MCID, thereby yielding a standardized metric expressed in ‘MCID units’. A proof‐of‐concept data set with seven simulated two‐arm studies was created to demonstrate the stepwise process of PROM prioritization, MCID‐based normalization and subsequent synthesis through random‐effects meta‐analysis using the restricted maximum likelihood (REML) estimator. Across the 100 included studies, 214 PROM mentions were identified, with modified Harris Hip Score (mHHS) (33.2%), International Hip Outcome Tool—12 items (iHOT‐12) (16.4%) and Hip Outcome Score—Sports Subscale (HOS‐SSS) (15.4%) being the most frequent. Normalization successfully transformed heterogeneous PROMs into a unified outcome scale, enabling synthesis of results as ‘number of clinically meaningful improvements’. The framework preserved clinical interpretability and allowed transparent, reproducible pooling of outcomes. A proof‐of‐concept data set of seven simulated studies illustrated stepwise MCID‐based normalization and synthesis through random‐effects meta‐analysis (REML). MCID normalization may offer a robust and clinically relevant methodology to harmonize diverse PROMs in HAS research. Its application may enhance comparability, reduce bias in evidence synthesis and support meaningful interpretation of meta‐analytic findings. Importantly, it translates heterogeneous PROMs into a unified, clinically interpretable metric, thereby helping clinicians and researchers to better assess treatment effectiveness across studies. This approach could also be adapted to other surgical fields with fragmented outcome reporting.

**Level of Evidence:**

Level V, expert opinion.

## Graphical abstract

MCID normalization: harmonizing heterogeneous PROMs in hip arthroscopy. Methodological proof‐of‐concept; simulated data set for illustration only.

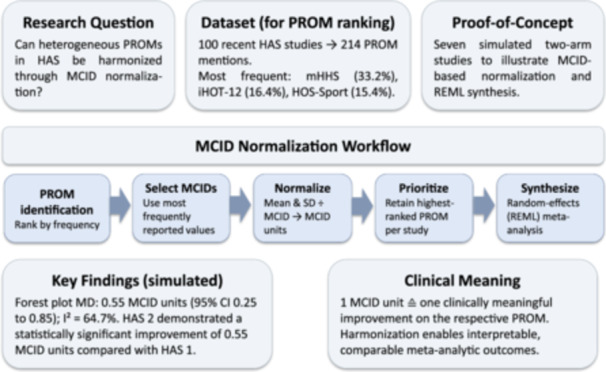

**Abbreviations**
CI: confidence interval
HAGOS: Copenhagen Hip and Groin Outcome Score
HAS: hip arthroscopy
HHS: Harris Hip Score
HOOS: Hip disability and Osteoarthritis Outcome Score
HOS‐ADL: Hip Outcome Score—Activities of Daily Living Subscale
HOS‐SSS: Hip Outcome Score—Sports Subscale
iHOT‐12: International Hip Outcome Tool—12 items
iHOT‐33: International Hip Outcome Tool—33 items
MCID: minimal clinically important difference
mHHS: modified Harris Hip Score
NAHS: Non‐Arthritic Hip Score
PROM: patient‐reported outcome measure
REML: restricted maximum likelihood
ROC: receiver operating characteristic
SD: standard deviation
WOMAC: Western Ontario and McMaster Universities Osteoarthritis Index

## INTRODUCTION

Patient‐reported outcome measures (PROMs) have progressively become the gold standard for evaluating patient benefit in orthopaedic procedures—particularly total hip and knee arthroplasty (THA/TKA)—owing to their direct capture of symptom relief, functional improvement and quality of life. Common instruments such as the Harris Hip Score (HHS) [39], Oxford Hip Score (OHS) [16] and modified Harris Hip Score (mHHS) [8] facilitate considerable standardization across studies and registries specializing in arthroplasty [97].

In contrast, in hip arthroscopy (HAS) a wide range of PROMs has been used in the literature, including Copenhagen Hip and Groin Outcome Score (HAGOS) [107], International Hip Outcome Tool—12 items (iHOT‐12)/International Hip Outcome Tool—33 items (iHOT‐33) [32, 63], Non‐Arthritic Hip Score (NAHS) [11], Hip disability and Osteoarthritis Outcome Score (HOOS) [72], Hip Outcome Score—Activities of Daily Living Subscale (HOS‐ADL)/Hip Outcome Score—Sports Subscale (HOS‐SSS) [58] and Western Ontario and McMaster Universities' Osteoarthritis Index (WOMAC) [3]. This heterogeneity likely reflects the absence of an established gold‐standard PROM in this relatively young field rather than true differences in underlying pathologies, thereby complicating cross‐study comparisons and meta‐analyses [104]. From a clinical perspective, this lack of standardization makes it difficult to judge treatment effectiveness across studies, hampers the formulation of evidence‐based guidelines and limits the ability of surgeons to compare outcomes when counselling patients.

To address the interpretative difficulties stemming from this heterogeneity, the concept of the minimal clinically important difference (MCID) has gained traction. MCID represents the smallest change in a PROM that patients perceive as beneficial. Specifically, in HAS literature, some studies have reported MCID for several PROMs offering interpretable thresholds of meaningful change [14, 59, 73]. However, although MCIDs provide valuable benchmarks, they have not yet been systematically operationalized to harmonize heterogeneous PROMs for meta‐analysis. This constitutes a major methodological and clinical research gap.

The present study proposes MCID‐based normalization as a systematic method for converting heterogeneous PROMs into a standardized metric of ‘MCID units’, enabling meta‐analyses that are both statistically valid and clinically interpretable. In essence, each PROM would be divided by its respective most frequently reported MCID, yielding a unified scale reflecting how many ‘clinically important improvements’ were achieved.

This study aimed to present and illustrate a robust methodological workflow for data preparation in systematic reviews and meta‐analyses of HAS outcomes, using MCID normalization to harmonize disparate PROMs and enhance methodological transparency, promote comparability across studies and ultimately support evidence synthesis that clinicians can interpret through a clinically meaningful lens. It was explored whether MCID normalization could facilitate the conversion of heterogeneous PROMs into a standardized and clinically interpretable metric suitable for evidence synthesis.

## METHODS

### Study design

This study is a methodological, nonclinical proof‐of‐concept work presenting a statistical framework for harmonizing heterogeneous PROMs in HAS meta‐analyses [82]. The approach was based on normalization of PROM values using their respective MCIDs. Data from previously published studies were extracted to identify and rank PROMs according to their frequency of reporting. To illustrate the workflow, a fully simulated data set of seven two‐arm studies was created solely for demonstration purposes. No clinical or patient‐level data were analysed. The design builds on previous systematic reviews and meta‐analyses that highlighted the challenges of PROM heterogeneity in hip surgery [90, 92, 93]. As no patient‐level data were included, ethical approval was not required.

#### Empirical analysis

##### PROM identification

Hip‐specific PROMs reported in the HAS literature were identified and ranked according to their frequency of use. To ensure methodological rigour and consistency, an approach similar to that described by Stone et al. [104] was followed. Therefore, a literature search was performed in PubMed up to 31 August 2025 using the search terms ((‘hip’) AND (‘arthroscopy’ OR ‘arthroscopic’ OR ‘endoscopy’ OR ‘endoscopic’ OR ‘preservation surgery’)). Only studies that could be clearly identified as hip arthroscopic or hip endoscopic procedures from the title were considered. Reviews, editorials, technical notes and other nonprimary studies were excluded. Among the eligible studies, the 100 most recently published primary publications reporting at least one functional outcome score in the abstract were included. This cut‐off was deliberately chosen to capture contemporary reporting patterns in HAS literature while maintaining methodological feasibility for manual data extraction and analysis. The aim was to establish a representative frequency distribution of PROM usage based on recent evidence rather than to conduct an exhaustive systematic review. From each of the 100 most recent studies, all relevant functional PROMs were extracted. As several studies reported more than one PROM, the total number of PROMs exceeded the number of included studies. The frequency of use was then counted to establish a ranking that guided PROM prioritization. For each included PROM, published MCID values were retrieved from the literature. In cases where multiple MCIDs were available, the most frequently reported value was selected, in line with prior methodological recommendations [14]. PROMs that were reported without specifying the version or subscale (e.g., iHOT without clarification or HOS without ADL/Sport differentiation) were excluded from the frequency analysis. In such cases, the study itself remained included, but only clearly specified PROMs were considered.

#### Recommended methodological framework

##### Data extraction

**Step 1:** Data on study characteristics (author, year, origin, sample size, design, patient characteristics, outcomes, follow‐up) and risk of bias should be extracted into a standardized spreadsheet.

**Step 2:** For quantitative data, both means and standard deviations (SDs) should be extracted whenever available.

**Step 3:** When SDs are not reported, they should be estimated from ranges using the formula:

$$\text{SD}\;=\left({\text{Range}}_{\max}{–\;\text{Range}}_{\min}\right)/4.$$

**Step 4:** If neither SDs nor ranges are available, missing SDs should be imputed according to established recommendations from the Cochrane Handbook [41].

##### PROM prioritization

**Step 1:** PROMs should be prioritized according to the ranking of frequency of use that was established in the empirical analysis of the 100 most recent HAS studies.

**Step 2:** The rationale is that the most commonly reported PROMs reflect prevailing practice and maximize comparability across studies, while reducing heterogeneity from rarely used instruments.

**Step 3:** When multiple PROMs are reported in a single study, the higher‐ranked PROM should be chosen for normalization, so that each cohort contributes one independent effect size without overweighting studies reporting several PROMs.

##### MCID normalization

**Step 1:** This step places all PROMs on a common, clinically interpretable metric, consistent with guidance on combining different instruments in meta‐analysis [82]. The prioritized PROM values should be transformed into a standardized ‘MCID unit’ scale by dividing observed means and SDs by the respective most frequently reported MCID of each score [14]:

$${\text{Value}}_{\text{MCID}}=\text{Observed}\;\text{Mean}\;/\;\text{MCID.}$$

**Step 2:** For changes over time, ‘change’ should be defined as the difference between postoperative and preoperative PROM values, reflecting the improvement after surgery in most cases. The following formula should be applied [41]:

$$\Delta_{\text{MCID}}=\left(\text{Postoperative}\;\text{Mean}\;–\;\text{Preoperative}\;\text{Mean}\right)\;/\;\text{MCID.}$$

**Step 3:** It should be noted that some PROMs are inversely scaled (i.e., higher values indicate worse outcomes). Therefore, careful attention should be paid to the definition provided in the primary study to ensure correct interpretation and normalization.

**Step 4:** To estimate the SD of the change score, it is important to note that the SD cannot simply be divided by the MCID, unlike mean values. Change scores represent paired data, and the corresponding variability must account for the correlation between pre‐ and postoperative measurements. Therefore, the following formula should be applied [67]:

$${\text{SD}}_{\Delta }=\surd \left({\text{SDpre}}^{\text{2}}+{\text{SDpost}}^{\text{2}}-2r\times \text{SDpre}\times \text{SDpost}\right)$$

where *r* denotes the correlation coefficient between pre‐ and postoperative values.

For comparability across instruments, this SD was then normalized by dividing by the PROM‐specific MCID:

$${\text{SD}}_{\Delta \text{mcid}}={\text{SD}}_{\Delta }/\text{MCID.}$$

#### Illustrative demonstration

##### Proof‐of‐concept application

To demonstrate the feasibility of the proposed framework, a synthetic, simulated data set was generated based on seven simulated two‐arm studies (Primary studies 1–7) comparing different HAS techniques (HAS 1 vs. HAS 2) for femoroacetabular impingement. Realistic PROM values reflecting the ranges commonly reported in the literature were used, but no clinical data were involved. The illustrative workflow included data extraction, transformation into MCID units and prioritization according to PROM frequency. These simulated data serve solely for methodological demonstration and are not derived from actual clinical trials.

### Statistical analysis

All functional primary outcomes were expressed in MCID units, where a value of 1.0 corresponds to one clinically important improvement, thereby providing a common metric across instruments [82]. This standardization rendered them suitable for data synthesis in meta‐analysis. In the simulated data set, mean differences (MDs) with 95% confidence intervals (CIs) were estimated for continuous outcomes (normalized functional MCID) using a frequentist random‐effects meta‐analysis with the restricted maximum likelihood (REML) heterogeneity estimator [41, 91]. Study weighting was performed with inverse variance. Statistical heterogeneity was assessed using Higgins' *I* ^2^ statistic, categorized as low (<25%), moderate (25%–75%) or high (>75%).

## RESULTS

### Literature search

**Figure 1:**
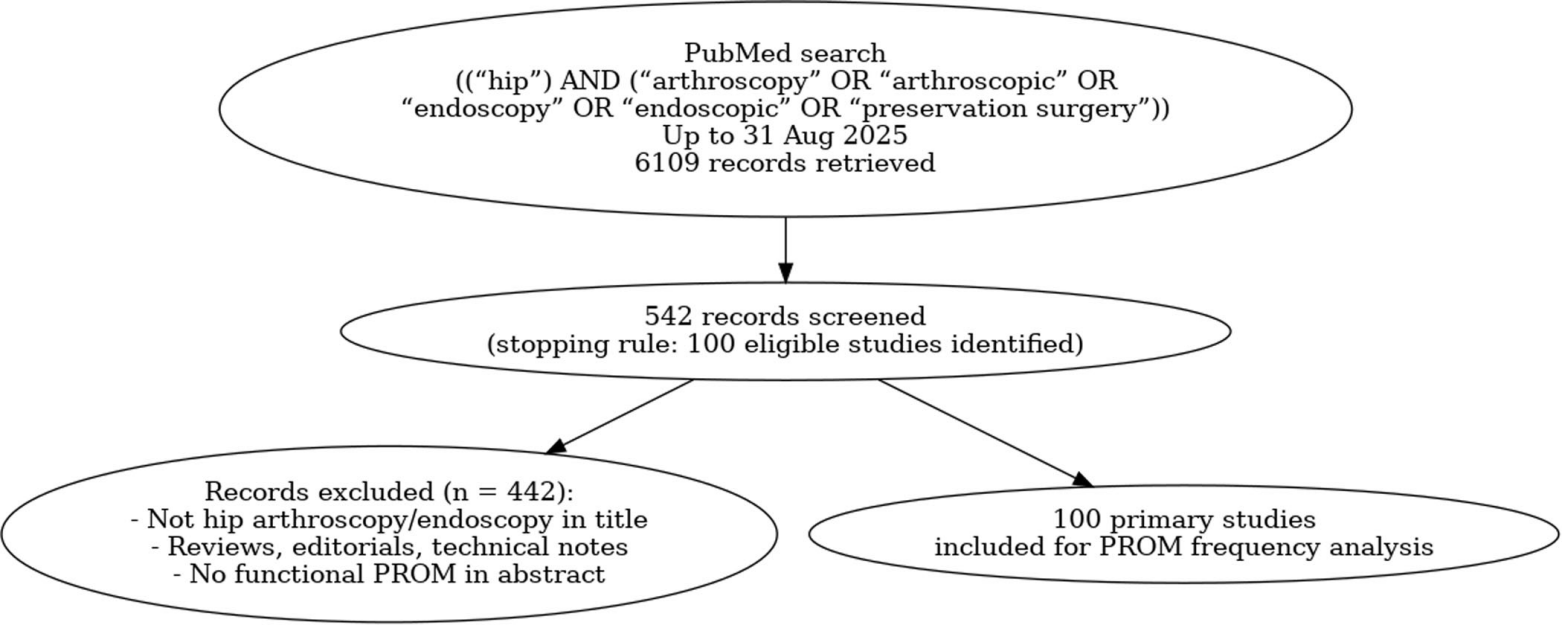
PRISMA‐style flowchart of the PubMed search and study selection process. A total of 6109 records were retrieved, 542 records were screened until 100 eligible primary studies were identified and ultimately included for PROM frequency analysis. PRISMA, Preferred Reporting Items for Systematic Reviews and Meta‐Analyses; PROM, patient‐reported outcome measures.

The PubMed search yielded 6109 records up to 31 August 2025. As the aim of this methodological study was to identify the most frequently reported PROMs rather than to perform a full systematic review, screening was stopped after 542 records. At this point, 100 most recently published eligible primary studies [1–2, 4–5, 7, 9,10,12.13, 15, 17–31, 33–38, 40, 42–57, 60–62, 64–66, 68–71, 74–81, 83–89, 94–96, 98–103, 105,106, 108–123] had been identified that fulfilled the inclusion criteria (HAS/hip endoscopy clearly indicated in the title and reporting at least one functional PROM in the abstract) (Figure 1). These 100 studies [1, 2, 4, 5, 7, 9, 10, 12, 13, 15, 17, 18, 19, 20, 21, 22, 23, 24, 25, 26, 27, 28, 29, 30, 31, 33, 34, 35, 36, 37, 38, 40, 42, 43, 44, 45, 46, 47, 48, 49, 50, 51, 52, 53, 54, 55, 56, 57, 60, 61, 62, 64, 65, 66, 68, 69, 70, 71, 74, 75, 76, 77, 78, 79, 80, 81, 83, 84, 85, 86, 87, 88, 89, 94, 95, 96, 98, 99, 100, 101, 102, 103, 105, 106, 108, 109, 110, 111, 112, 113, 114, 115, 116, 117, 118, 119, 120, 121, 122, 123] were then analysed for frequency of PROM reporting (Table 1).

**Table 1:** Included studies and reported PROMs.

	Study	iHOT‐12	mHHS	iHOT‐33	HOS‐SSS	HOS‐ADL	NAHS	HOOS^a^	WOMAC	HAGOS^a^	HHS
1.	Gürsan O et al. [37]		X								
2.	Yoshitani J et al. [117]	X									
3.	Martin‐Lozoya J et al. [57]			X	X	X					
4.	Johnson BT et al. [46]		X								
5.	Quesada‐Jimenez R et al. [87]	X	X		X		X				
6.	Bodine BG et al. [7]		X								
7.	Jia D et al. [43]		X								
8.	Gillinov SM et al. [29]		X								
9.	Baldwin RB et al. [2]	X	X								
10.	Zhang HS et al. [118]	X	X				X				
11.	Wang Y et al. [111]	X	X					X			
12.	Comfort SM et al. [13]		X		X	X	X				
13.	Quesada‐Jimenez R et al. [88]		X		X		X				
14.	Paliobeis AS et al. [74]		X								
15.	Zheng F et al. [119]		X								
16.	Ruzbarsky JJ et al. [99]		X		X	X			X		
17.	Kantor AH et al. [47]	X	X								
18.	Guo ZK et al. [35]	X	X								
19.	Berzolla E et al. [4]		X				X				
20.	Teng HR et al. [106]	X	X								
21.	Jia Q et al. [44]	X	X		X	X					
22.	Ma JP et al. [54]	X	X								
23.	He Y et al. [40]	X	X								
24.	Quesada‐Jimenez R et al. [86]		X		X		X				
25.	Danilkowicz RM et al. [15]	X	X		X	X					
26.	Park CW et al. [76]		X								
27.	Quesada‐Jimenez R et al. [83]	X	X		X		X				
28.	Gosnell GG et al. [31]		X				X				
29.	Nikou S et al. [70]	X									
30.	Erlandsson D et al. [20]									X	
31.	Maldonado DR et al. [56]	X	X		X		X				
32.	Quesada‐Jimenez R et al. [89]		X		X		X				
33.	Mullins K et al. [68]		X								
34.	Gahleitner M et al. [24]							X			
35.	Poutre RL et al. [81]		X	X	X	X					
36.	Kwiecien SY et al. [49]		X							X	
37.	Gilat R et al. [28]	X	X		X	X					
38.	Golden MV et al. [30]		X		X	X					
39.	Perraut G et al. [79]		X								
40.	Dean MC et al. [17]			X							
41.	Winzenried AE et al. [113]	X			X	X					
42.	Filan D et al. [21]		X								
43.	Moran TE et al. [64]	X	X		X	X					
43.	Lee JS et al. [51]		X	X	X	X	X				
44.	Morgan AM et al. [66]		X								
45.	Foo GL et al. [23]	X					X	X			
46.	Aprato A et al. [1]		X								
47.	Berzolla E et al. [5]		X				X				
48.	Vera AM et al. [109]				X	X					
49.	Mullins K et al. [69]		X						X		
50.	Quesada‐Jimenez R et al. [84]		X		X		X				
51.	Siddiq BS et al. [102]		X	X	X	X	X				
52.	Xia KY et al. [115]					X	X				X
53.	Matsushita Y et al. [62]	X									
54.	Ruff G et al. [98]		X				X				
55.	Gilat R et al. [27]		X		X	X					
56.	Güven Ş et al. [38]		X								
57.	Cervantes JE et al. [9]	X	X		X	X					
58.	Shen LY et al. [100]		X		X	X					
59.	Spencer AD et al. [103]							X			
60.	Tassinari E et al. [105]							X			
61.	Marty EW et al. [61]	X					X				
62.	Richey AE et al. [96]							X			
63.	Domb BG et al. [18]		X		X		X				
64.	Jan K et al. [42]	X			X	X					
65.	Filan D et al. [22]		X								
67.	Uzun E et al. [108]		X						X		
68.	Pepic L et al. [78]							X			
69.	Gao G et al. [26]		X								
70.	Yang D et al. [116]		X								
71.	Quesada‐Jimenez R et al. [85]	X	X								
72.	Morgan AM et al. [65]		X				X				
73.	Zhu Y et al. [120]	X	X				X				
74.	Palmer A et al. [75]					X					
75.	Maldonado DR et al. [55]				X	X					
76.	Richards SM et al. [95]		X		X						
77.	Lee JH et al. [50]	X					X				
78.	Gao G et al. [25]		X								
79.	Zhu Y et al. [121]	X	X								
80.	Karlsson L et al. [48]	X								X	
81.	Guillaume G et al. [33]	X	X				X				
82.	Nikou S et al. [71]	X								X	
83.	Zhu Y et al. [122]		X								
84.	Lv Y et al. [53]		X		X	X					
85.	Wu W et al. [114]										X
86.	Wilson H et al. [112]	X									
87.	Lee JS et al. [52]		X	X	X	X					
88.	Gou Y et al. [34]	X	X			X					
89.	Jochimsen KN et al. [45]	X									
90.	Randelli F et al. [94]				X	X	X				
91.	Çiçeklidağ M et al. [12]		X								
92.	Dornan GJ et al. [19]		X			X			X		
93.	Shimizu K et al. [101]	X	X		X		X				
94.	Champagne G et al. [10]						X				
95.	Pepic L et al. [77]							X			
96.	Guo Z et al. [36]		X								
97.	Zhu Y et al. [123]	X	X				X				
98.	Martin SD et al. [60]		X	X							
99.	Vogel MJ et al. [110]				X	X					
100.	Philippon MJ et al. [80]		X		X	X					

*Note*: Overview of the 100 included primary studies and their reported hip‐specific PROMs. Each ‘X’ indicates that the corresponding PROM was reported in the respective study.

Abbreviations: HAGOS, Copenhagen Hip and Groin Outcome Score; HHS, Harris Hip Score; HOOS, Hip disability and Osteoarthritis Outcome Score; HOS‐ADL, Hip Outcome Score—Activities of Daily Living Subscale; HOS‐SSS, Hip Outcome Score—Sports Subscale; iHOT‐33, International Hip Outcome Tool—33 items; mHHS, modified Harris Hip Score; PROMs, patient‐reported outcome measures; iHOT‐12, International Hip Outcome Tool—12 items; NAHS, Non‐Arthritic Hip Score; WOMAC, Western Ontario and McMaster Universities Osteoarthritis Index.

^a^ Any mention of HOOS and HAGOS was counted (full scale, any subscale or short forms). Reporting was heterogeneous and often unspecified; functional subscales were seldom reported.

### PROM identification and prioritization

**Figure 2:**
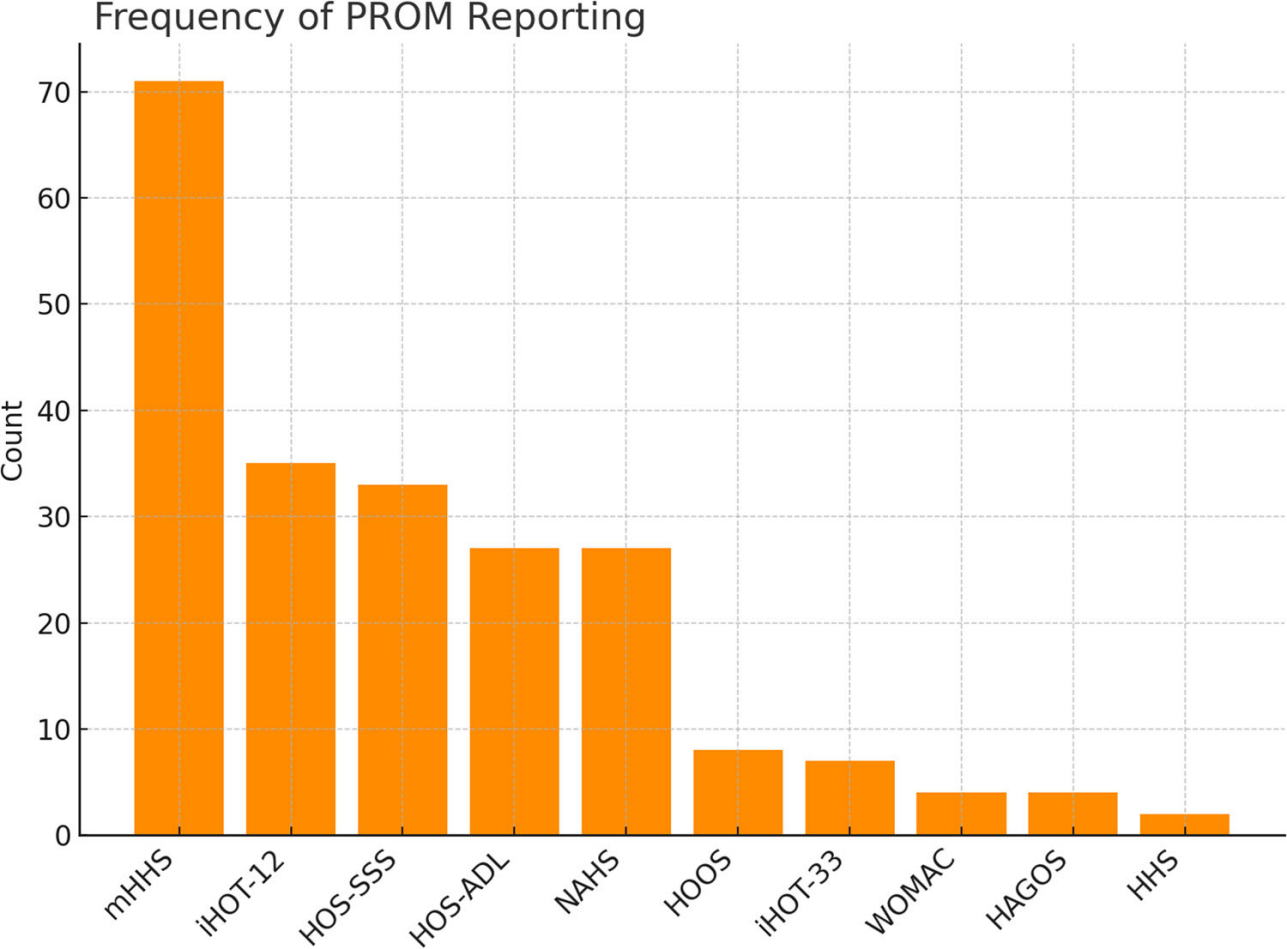
Frequency of hip‐specific PROMs reported in 100 included HAS studies. The mHHS was most frequently reported, followed by the iHOT‐12, HOS‐SSS, NAHS and HOS‐ADL. Less frequently used instruments included HOOS, iHOT‐33, WOMAC, HAGOS and HHS. HAGOS, Copenhagen Hip and Groin Outcome Score; HAS, hip arthroscopy; HHS, Harris Hip Score; HOS‐ADL, Hip Outcome Score—Activities of Daily Living Subscale; HOS‐SSS, Hip Outcome Score—Sports Subscale; HOOS, Hip disability and Osteoarthritis Outcome Score; iHOT‐12, International Hip Outcome Tool—12 items; iHOT‐33, International Hip Outcome Tool—33 items; mHHS, modified Harris Hip Score; NAHS, Non‐Arthritic Hip Score; PROM, patient‐reported outcome measures; WOMAC, Western Ontario and McMaster Universities Osteoarthritis Index.

In total, 100 primary studies [1, 2, 4, 5, 7, 9, 10, 12, 13, 15, 17, 18, 19, 20, 21, 22, 23, 24, 25, 26, 27, 28, 29, 30, 31, 33, 34, 35, 36, 37, 38, 40, 42, 43, 44, 45, 46, 47, 48, 49, 50, 51, 52, 53, 54, 55, 56, 57, 60, 61, 62, 64, 65, 66, 68, 69, 70, 71, 74, 75, 76, 77, 78, 79, 80, 81, 83, 84, 85, 86, 87, 88, 89, 94, 95, 96, 98, 99, 100, 101, 102, 103, 105, 106, 108, 109, 110, 111, 112, 113, 114, 115, 116, 117, 118, 119, 120, 121, 122, 123] met the inclusion criteria and were analysed for frequency of PROM reporting. Across these studies, 214 PROM mentions were identified (Table 2 and Figure 2). The mHHS [8] was by far the most frequently reported outcome measure (71 mentions, 33.2%), followed by the iHOT‐12 (35 mentions, 16.4%) [32], the HOS‐Sport Subscale (HOS‐SSS) (33 mentions, 15.4%) [58], the NAHS (27 mentions, 12.6%) [11] and the HOS‐ADL (27 mentions, 12.6%) [58]. Other PROMs such as HOOS [72], iHOT‐33 [63], WOMAC [3] and HAGOS [107] were reported less frequently (≤8%). Rarely used instruments included the original HHS [39] (two mentions, 0.9%). Any mention of HOOS and HAGOS was counted (full scale, any subscale or short forms). Reporting was heterogeneous and often unspecified; functional subscales were seldom reported.

**Table 2:** Frequency of PROM reporting.

PROM	Count	Percent
mHHS	71	32.6
iHOT‐12	35	16.1
HOS‐SSS	33	15.1
HOS‐ADL	27	12.4
NAHS	27	12.4
HOOS^a^	8	3.7
iHOT‐33	7	3.2
WOMAC	4	1.8
HAGOS^a^	4	1.8
HHS	2	0.9

Abbreviations: HAGOS, Copenhagen Hip and Groin Outcome Score; HHS, Harris Hip Score; HOOS, Hip disability and Osteoarthritis Outcome Score; HOS‐ADL, Hip Outcome Score—Activities of Daily Living Subscale; HOS‐SSS, Hip Outcome Score—Sports Subscale; iHOT‐12, International Hip Outcome Tool—12 items; iHOT‐33, International Hip Outcome Tool—33 items; mHHS, modified Harris Hip Score; NAHS, Non‐Arthritic Hip Score; PROM, patient‐reported outcome measure; WOMAC, Western Ontario and McMaster Universities Osteoarthritis Index.

a Any mention of HOOS and HAGOS was counted (full scale, any subscale or short forms). Reporting was heterogeneous and often unspecified; functional subscales were seldom reported.

### MCID normalization

To enable harmonization across heterogeneous outcome measures, all PROMs were transformed into standardized MCID units. Table 3 summarizes the most frequently reported MCID values applied for each of the ten most frequently reported hip‐specific PROMs in HAS research. The selected values were derived from prior validation or methodological studies and represent the most frequently reported thresholds available, thereby ensuring comparability across studies. The MCID was set at 9.5 points for mHHS [73], 13.0 points for iHOT‐12 [59, 73], 12.1 points for HOS‐SSS [73], 8.5 points for NAHS [6], 9.8 points for HOS‐ADL [73] and 10.7 points for iHOT‐33 [73]. These values were used to normalize both mean scores and SDs, facilitating direct comparison of functional outcomes across otherwise heterogeneous PROMs.

**Table 3:** Most frequently reported MCID values for key PROMs.

Score	Population/Setting	Conservative MCID	Typical scale	Primary source (first author, year)	DOI	Reference
mHHS	Hip arthroscopy	9.5	0–100	Nwachukwu BU, 2018	doi:10.1016/j.arthro.2018.01.050	[73]
iHOT‐12	Hip arthroscopy	13.0	0–100	Nwachukwu BU, 2018: Martin RL, 2019	doi:10.1016/j.arthro.2018.01.050; doi:10.1016/j.arthro.2018.09.028	[59, 73]
HOS‐SSS	Hip arthroscopy	12.1	0–100	Nwachukwu BU, 2018	doi:10.1016/j.arthro.2018.01.050	[73]
NAHS	Hip arthroscopy	8.5	0–100	Bloom DA, 2022	doi:10.1007/s00167‐021‐06756‐9	[6]
HOS‐ADL	Hip arthroscopy	9.8	0–100	Nwachukwu BU, 2018	doi:10.1016/j.arthro.2018.01.050	[73]
iHOT‐33	Hip arthroscopy	10.7	0–100	Nwachukwu BU, 2018	doi:10.1016/j.arthro.2018.01.050	[73]

*Note*: The most frequently reported MCID values for the most frequently used hip‐specific PROMs.

Abbreviations: HOS‐ADL, Hip Outcome Score—Activities of Daily Living Subscale; HOS‐SSS, Hip Outcome Score—Sports Subscale; iHOT‐12, International Hip Outcome Tool—12 items; iHOT‐33, International Hip Outcome Tool—33 items; MCID, minimal clinically important difference; mHHS, modified Harris Hip Score; NAHS, Non‐Arthritic Hip Score; OA, osteoarthritis; PROMs, patient‐reported outcome measures.

### Proof‐of‐concept application

As a proof of concept, seven simulated two‐arm primary studies (Primary studies 1–7) comparing two HAS techniques (HAS 1 vs. HAS 2) were constructed to demonstrate the stepwise application of the MCID normalization framework.

Table 4 displays the extracted PROM values, already ordered according to the previously established prioritization ranking. For each PROM, the corresponding most frequently reported MCID values are provided, but PROMs and MCIDs have not yet been mathematically converted at this stage.

**Table 4:** Extracted PROMs from simulated studies (prenormalization).

Primary study	Procedure	Patients	Postoperative mHHS		Postoperative iHOT‐12		Postoperative HOS‐SSS		Postoperative NAHS		Postoperative HOS‐ADL		Postoperative iHOT‐33	
			MCID = 9.5		MCID = 13.0		MCID = 12.1		MCID = 8.5		MCID = 9.8		MCID = 10.7	
	1 = HAS 1													
	2 = HAS 2													
		*N*	Points	SD	Points	SD	Points	Points	Points	SD	Points	SD	Points	SD
Primary study 1	1	22	84.7	11.4	81.2	12.6								
	2	21	84.0	12.5	77.6	15.7								
Primary study 2	1	177											76.4	16.8
	2	171											70.8	18.8
Primary study 3	1	50									91.3	9.2		
	2	49									88.0	10.8		
Primary study 4	1	40							82.0	13.3	86.3	11.3		
	2	40							79.8	14.3	84.8	12.9		
Primary study 5	1	46			75.5	11.2	80.5	16.0			82.8	13.6		
	2	44			69.6	13.1	73.1	18.3			80.1	14.8		
Primary study 6	1	45			75.6	12.5								
	2	46			64.4	15.4								
Primary study 7	1	110									78.8	15.4		
	2	112									75.9	16.5		

*Note*: Extracted PROMs from seven simulated primary studies (HAS 1 vs. HAS 2), ordered according to the established frequency‐based prioritization. The most frequently reported MCID values are listed for each PROM, but no conversion to MCID units has been performed at this stage.

Abbreviations: HAS, hip arthroscopy; HOS‐ADL, Hip Outcome Score—Activities of Daily Living Subscale; HOS‐SSS, Hip Outcome Score—Sports Subscale; HOOS, Hip disability and Osteoarthritis Outcome Score; iHOT‐12, International Hip Outcome Tool—12 items; iHOT‐33, International Hip Outcome Tool—33 items; MCID, minimal clinically important difference; mHHS, modified Harris Hip Score; NAHS, Non‐Arthritic Hip Score; PROMs, patient‐reported outcome measures; SD, standard deviation.

In Table 5, both mean PROM values and SDs were transformed into MCID units. Further, this table illustrates the prioritization process. For each of the seven primary studies, one PROM was retained based on the frequency ranking to create a unified functional MCID outcome. The retained values are highlighted in green, whereas nonselected PROMs are indicated in red, showing how less frequently used instruments were excluded once a higher‐ranked PROM was available.

**Table 5:** PROM means and SDs from the simulated data set converted into MCID units.

Primary study	Procedure	Patients	Postoperative mHHS		Postoperative iHOT‐12		Postoperative HOS‐SSS		Postoperative NAHS		Postoperative HOS‐ADL		Postoperative iHOT‐33		Postoperative functional MCID	
			MCID = 9.5		MCID = 13.0		MCID = 12.1		MCID = 8.5		MCID = 9.8		MCID = 10.7			
	1 = HAS 1															
	2 = HAS 2															
		*N*	Points	SD	Points	SD	Points	SD	Points	SD	Points	SD	Points	SD	Points	SD
Primary study 1	1	22	8.9	1.2	6.2	1.0									8.9	1.2
	2	21	8.8	1.3	6.0	1.2									8.8	1.3
Primary study 2	1	177											7.1	1.6	7.1	1.6
	2	171											6.6	1.8	6.6	1.8
Primary study 3	1	50									9.3	0.9			9.3	0.9
	2	49									9.0	1.1			9.0	1.1
Primary study 4	1	40							9.6	1.6	8.8	1.2			9.6	1.6
	2	40							9.4	1.7	8.7	1.3			9.4	1.7
Primary study 5	1	46			5.8	0.9	6.7	1.3			8.4	1.4			5.8	0.9
	2	44			5.4	1.0	6.0	1.5			8.2	1.5			5.4	1.0
Primary study 6	1	45			5.8	1.0									5.8	1.0
	2	46			5.0	1.2									5.0	1.2
Primary study 7	1	110									8.0	1.6			8.0	1.6
	2	112									7.7	1.7			7.7	1.7

*Note*: Prioritization of PROMs across the seven simulated primary studies based on the frequency ranking. Grey values represent the retained PROMs contributing to the unified functional MCID outcome, while red values indicate nonselected PROMs that were excluded due to lower priority.

Abbreviations: HAS, hip arthroscopy; HOS‐ADL, Hip Outcome Score—Activities of Daily Living Subscale; HOS‐SSS, Hip Outcome Score—Sports Subscale; iHOT‐12, International Hip Outcome Tool—12 items; iHOT‐33, International Hip Outcome Tool—33 items; MCID, minimal clinically important difference; mHHS, modified Harris Hip Score; NAHS, Non‐Arthritic Hip Score; PROMs, patient‐reported outcome measures; SD, standard deviation.

**Figure 3:**
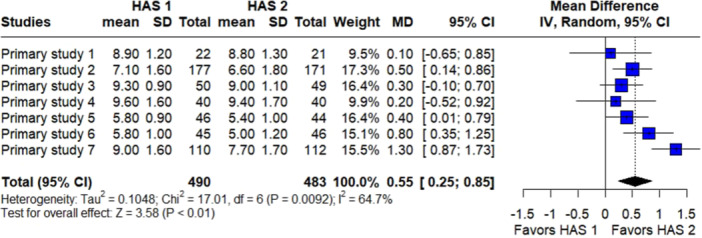
Forest plot of simulated data (proof‐of‐concept demonstration). Forest plot of the unified functional MCID outcomes derived from seven simulated primary studies (HAS 1 vs. HAS 2), demonstrating how heterogeneous PROMs can be harmonized on a common, clinically interpretable scale for meta‐analysis. CI, confidence interval; HAS, hip arthroscopy; MCID, minimal clinically important difference; PROMs, patient‐reported outcome measure; SD, standard deviation.

Finally, Figure 3 presents the forest plot synthesizing the normalized functional MCID outcomes from Primary studies 1–7. The plot demonstrates how heterogeneous PROMs can be harmonized into a single, interpretable summary metric for meta‐analysis. HAS 2 demonstrated a statistically significant improvement of 0.55 MCID units compared with HAS 1 (MD = 0.55 MCID units; 95% CI: 0.25–0.85; *I* ^2^ = 64.7%) (Figure 3). Given that 1 MCID unit represents a clinically meaningful improvement on the respective PROM scale, this difference indicates that patients treated with HAS 2 achieved, on average, slightly greater functional gains that were both statistically and clinically relevant. The moderate heterogeneity reflects the variability intentionally embedded in the simulated data set to illustrate the framework's analytical properties rather than true clinical diversity.

## DISCUSSION

This methodological study demonstrates a systematic framework to harmonize heterogeneous PROMs in HAS through MCID normalization. To my knowledge, this represents one of the first structured workflows aiming to translate PROM diversity into a clinically interpretable, unified outcome metric. The justification for proposing this framework lies in the author's established expertise in hip surgery meta‐analyses, where PROM heterogeneity repeatedly emerged as a barrier to evidence synthesis.

The proof‐of‐concept application shows that different PROMs can be transformed into MCID units, prioritized and synthesized without sacrificing interpretability. This step addresses a critical gap in the field: while MCIDs are widely studied, they are rarely operationalized to solve incompatibility between scores. By anchoring results in MCID units, meta‐analyses can report pooled improvements in ‘number of clinically meaningful changes’, which is directly interpretable to clinicians and patients.

The framework provides a reproducible tool for future systematic reviews and clinical trials. It enhances comparability, enables more robust pooled estimates and offers a clinically meaningful interpretation. Beyond HAS, the approach can be adapted to other surgical fields with fragmented outcome reporting. To facilitate application of the proposed methodology, a factbox summarizing the stepwise framework for MCID normalization is presented in Table 6.

**Table 6:** FACTBOX—stepwise framework for MCID normalization.

**Identify PROMs** Extract all hip‐specific PROMs reported in included studies. Rank PROMs according to frequency of use in the literature.
**Select MCID values** Retrieve published MCIDs for each PROM. Choose the most frequently reported or widely accepted value. Check scale direction: Some PROMs are inverted—verify definitions in the primary. study before normalization.
**Normalize PROMs** Convert means and SDs into MCID units: ValueMCID = Observed value ÷ MCID ΔMCID = (Postop – Preop) ÷ MCID SDΔMCID = √(SDpre^2^ + SDpost^2^ – 2*r* × SDpre × SDpost) ÷ MCID
**Prioritize PROMs** When multiple PROMs are reported per study, retain only the highest‐ranked PROM. Discard lower‐ranked PROMs to avoid statistical dependence.
**Synthesize outcomes** Meta‐analyse the unified functional MCID outcomes. Express results as ‘number of clinically important improvements’.
**Interpret findings** Report pooled outcomes in MCID units, directly interpretable for clinicians and patients.

Abbreviations: MCID, minimal clinically important difference; PROMs, patient‐reported outcome measure; SD, standard deviation.

Recent evidence illustrates why harmonization is necessary: in a large foot‐and‐ankle registry, Zona et al. demonstrated that Patient‐Reported Outcomes Measurement Information System (PROMIS)‐based MCIDs vary substantially by surgery type but remain relatively stable across demographics, suggesting that a single global MCID can misrepresent clinically meaningful change [124]. Moreover, Puhan et al. demonstrated that instruments can be highly correlated yet differ substantially in responsiveness, cautioning against naïve pooling and supporting the present framework's use of instrument‐specific, frequently reported MCIDs and a single‐instrument rule [82].

An important aspect of this work is its clinical applicability. By expressing heterogeneous PROMs in terms of MCID units, the framework translates statistical outcomes into a clinically meaningful scale that reflects the number of patients achieving a relevant improvement. This allows surgeons to better interpret pooled results, facilitates patient counselling and supports the development of evidence‐based treatment guidelines. Moreover, the methodology is not restricted to HAS but can be generalized to other fields where outcome reporting is fragmented.

Key strengths include the explicit methodological workflow, literature‐based PROM prioritization and the use of the most frequently reported MCID values. Limitations include reliance on published MCIDs, which may vary across populations, surgical indications and follow‐up times, potentially affecting comparability between studies. Furthermore, the illustrative proof‐of‐concept data set was simulated and not derived from real trials. Furthermore, the choice of most frequently reported MCIDs may underestimate improvements but ensures methodological rigour. MCID normalization aligns scales but not constructs; residual differences between PROM content persist despite harmonization. Another limitation is that published MCID values were derived using different methods (anchor‐, distribution‐, receiver operating characteristic [ROC]‐based or predefined thresholds), which were not consistently reported. This methodological heterogeneity across PROMs should be considered when interpreting normalized results.

## CONCLUSION

MCID normalization offers a transparent and clinically interpretable framework to harmonize heterogeneous PROMs in HAS. Adoption of this approach may improve the quality and comparability of future meta‐analyses in the field. Clinically, this framework enables more comparable and interpretable outcome synthesis in HAS, supporting patient counselling and guideline development.

## AUTHOR CONTRIBUTIONS

Nikolai Ramadanov performed the entire work.

## CONFLICT OF INTEREST STATEMENT

The author declares no conflicts of interest.

## ETHICS STATEMENT

The author has nothing to report.

## Data Availability

The raw data extraction set is provided for the main text.
